# Biodiesel Purification by Solvent-Aided Crystallization Using 2-Methyltetrahydrofuran

**DOI:** 10.3390/molecules28031512

**Published:** 2023-02-03

**Authors:** Wan Nur Aisyah Wan Osman, Nur Athirah Izzati Badrol, Shafirah Samsuri

**Affiliations:** 1 Chemical Engineering Department, Universiti Teknologi PETRONAS, Seri Iskandar 32610, Malaysia; 2 HICoE-Centre for Biofuel and Biochemical Research (CBBR), Institute of Sustainable Buiding, Universiti Teknologi PETRONAS, Seri Iskandar 32610, Malaysia

**Keywords:** 1-butanol, 2-methyltetrahydrofuran, biodiesel, coolant temperature, cooling time, green solvent, stirring speed, solvent-aided crystallization

## Abstract

The previous biodiesel purification by Solvent-Aided Crystallization (SAC) using 1-butanol as assisting agent and parameters for SAC were optimized such as coolant temperature, cooling time and stirring speed. Meanwhile, 2-Methyltetrahydrofuran (2-MeTHF) was selected as an alternative to previous organic solvents for this study. In this context, it is used to replace solvent 1-butanol from a conducted previous study. This study also focuses on the technological improvements in the purification of biodiesel via SAC as well as to produce an even higher purity of biodiesel. Experimental works on the transesterification process to produce crude biodiesel were performed and SAC was carried out to purify the crude biodiesel. The crude biodiesel content was analyzed by using Gas Chromatography–Mass Spectrometry (GC-MS) and Differential Scanning Calorimetry (DSC) to measure the composition of Fatty Acid Methyl Esters (FAME) present. The optimum value to yield the highest purity of FAME for parameters coolant temperature, cooling time, and stirring speed is −4 °C, 10 min and 210 rpm, respectively. It can be concluded that the assisting solvent 2-MeTHF has a significant effect on the process parameters to produce purified biodiesel according to the standard requirement.

## 1. Introduction

Nowadays, the depletion of fossil resources is not something new as the global population keeps on rising. This has led to the discovery of renewable fuels, such as biodiesel. Biodiesel has attracted a lot of interest as a future fuel because of its copious resources and environmental considerations [1]. The bio-based fuel business has seen an accelerated surge in sales and has become a driving force to create novel green technologies. These were influenced by government laws and concerns about ecological sustainability and the depletion of natural raw materials. Biodiesel’s initial design was careful and methodical, emphasizing the industry in terms of long-term viability. Nowadays, this biofuel is easy to integrate into existing facilities and cars, and the industry sector has devoted a lot of effort to researching and promoting the fuel’s capabilities.

In Malaysia, fossil fuels accounted for 95% of the overall primary energy output in the year 2006 [2]. This includes natural gas, petroleum, coal, peat renewables, and hydroelectricity. Primary energy is generally raw energy that has not been engineered or converted in any way. Malaysia is presently a fast-expanding country; thus, this prevalent tendency is likely to continue speculating for the next 20 years. On top of that, the study also claimed that Malaysia is presently the world’s largest exporter of palm oil, despite being the oil’s second-biggest producer after Indonesia [2]. On that account, Malaysia endeavoured to gain leverage in the expanding biofuel sector by encouraging palm oil-based biodiesel development upon recognizing its profitability. Due to this, Malaysia has been recognized as one of the countries that proactively encourages commercial operations for the use of biodiesel as a fossil fuel substitute [2].

The authors also stated that the biodiesel sector in Malaysia shows no activity until the Eighth Malaysia Plan, in the year 2001, established the Fifth Fuel Policy [2]. Renewable energy has been designated as the fifth source of electricity generation in Malaysia under the proposed legislation. The Malaysian biodiesel sector is also largely supported by the National Biofuel Policy. The legislation concentrates on biodiesel commercialization, utilization, study, development, and exportation, yet it excludes upstream parts of the industry growth. Biodiesel production and deployment are expected to keep on increasing, particularly in rapidly developing countries where economic development is accelerating. Malaysia expects to supply one million tonnes of biodiesel by the end of 2020, increasing 80% production compared to the previous year (2019) [1].

The process of separating contaminants from biodiesel is crucial to ensure that the developed fuel fulfils all required standards, delivering improved performance as well as preserving the engine from degradation [3]. Glycerol, soap, water, a catalyst used, and triglycerides are mostly residues that must be separated from crude biodiesel obtained. Purification is known to be one of the most essential stages in biodiesel production. Water washing, ion exchange adsorbents, and membrane-based adsorbents are the foremost often utilized technologies for the purification of biodiesel [4]. This purification method is critical in maintaining efficacy in engine performance. According to Arenas et al. [4], free fatty acids at high concentrations can develop deposit accounts in storage tanks and even injectors, hence reducing the lifespan of engines. In addition, the high water content can corrode the engine of automobiles. Therefore, the purification of crude biodiesel can be challenging as it contributes to the rise in biodiesel operating expenses. This opens up a discussion on the possible alternatives to the conventional method of biodiesel purification.

Purification of biodiesel is undoubtedly one of the important steps in biodiesel production. The main goal of the production process is to achieve high-quality fuel with hardly any contaminants that could sabotage its excellence. The impurities that could be present in biodiesel are glycerol, alcohol (namely, methanol), soap, free fatty acids, residual salts, metals, and production catalysts [5]. It is clear that the densities of biodiesel and glycerol are disparate enough to have them separated by gravitational settling and centrifugation [6]. Having different polarities is another determinant on the account that the separation between the ester and glycerol is rapid. Glycerol must be purified as it contains a large part of biodiesel impurities, and it would deposit at the bottom of the fuel tank causing the fouling of the injector [7]. The complete elimination of glycerol represents the exceptional quality of biodiesel. Another polar substance, methanol, is necessary to be removed as it has a low flash point which can be an inconvenience in terms of transportation, storage, and utilization [7]. In addition, they also mentioned that methanol is also a result of corrosion to pieces of aluminium and zinc [7].

Various techniques have been applied for the application of biodiesel purification in order to overcome the limitation of high water usage on the earlier method explained. Recently, a new method had been introduced known as SAC. This method is carried out under low temperatures compared to other biodiesel purification techniques. Hence, it could prevent the biodiesel from becoming volatile during or after the purification process. This is supported by studies mentioning that the biodiesel would be volatile at higher temperatures, in the range of 340–375 °C, which were obtained from thermal analyses of thermogravimetric analysis (TGA) and differential scanning calorimetry (DSC) [8,9]. 

The basic principle of SAC is to selectively reduce the viscosity of melts to alter the crystallization kinetics by the insertion of assisting agent with adequate quantities into the solvents [10]. Once the assistant solvents are injected, rapid crystallization occurs in a low-viscosity sample solution. This method is able to overcome the biggest difficulty in separating biodiesel–glycerol, where both are hard to separate [11]. This is due to these solvents creating high-viscosity crude melts that are difficult to distinguish by conventional methods, which had appealed to a notion that permits layer crystallization to extract these compounds.

Samsuri et al. [12] concluded that SAC could effectively remove undesired glycerol, methanol, and soap components, leaving a sample obtained known as purified biodiesel. Thus, it is an operative practice for a waterless approach to refine biodiesel in a more ecologically friendly way than other common purifying procedures, while being able to reduce the cost required for wastewater treatment afterwards. As a result, it is indeed critical to evaluate whether it is feasible for a certain solvent to be appropriate for each system besides not knowing the effects of crystallization during operation. Recent findings showed that SAC is highly influenced by the following parameters: concentration of solvent, cooling temperature and time, and stirring rate [12]. The optimum parameter is obtained by using the analysis technique of response surface plot analysis. Surface plots can be used to evaluate targeted response values and the connection of the operational parameters. It is found that biodiesel with a purity of 99.375% is obtained as the optimum condition by using the following parameters: concentration of solvent of 1.5 wt%, cooling temperature of 12.7 °C, cooling time of 35 min and stirring rate of 175 rpm. However, this study used 1-butanol as the assisting solvent. 

1-butanol has a poor separation performance as an assisting solvent for SAC. This statement had been proven by Ahmad and Samsuri [11]. They analyzed the effect of different concentrations of 1-butanol in order to evaluate the optimum quantity of 1-butanol required for the biodiesel purification process via SAC. They used ultrasonic irradiation to aid this process and findings showed that the purity of biodiesel reduced as the concentration of 1-butanol increased. Conversely, inadequate 1-butanol could cause impure crystals forming resulting in nucleation, where the crystals might form alongside the whole chemical freezes. Therefore, they claimed that high-purity biodiesel may be achieved at lower cooling temperatures and intermediary 1-butanol concentrations, or with a longer response time if excess 1-butanol is employed.

Therefore, sustainable solution by using green alternatives in the purification process has been studied and researched to gain biodiesel satisfactory with its standard to lessen the ecological implications of using solvents in chemical processing. The use of environmentally sustainable solvents or green alternatives to traditional goods has recently gained a lot of interest, citing environmental advantages and worker safety as reasons. Green chemistry had been introduced as a way in managing effluent produced from chemical processes, specifically from the processing industry [13]. The sole purpose is to focus on the environmental effect of chemistry and eradicate environmental pollution through concerted, long-term preventative efforts. This concept led to the proposal of a low-toxicity alternative solvent with broad synthetic applications for the processing sector. 

In this experiment, solvent 1-butanol is substituted with 2-Methyltetrahydrofuran (2-MeTHF) as an alternative assisting agent for crystallization and a better replacement in terms of environmental aspects for the said organic solvent. 2-MeTHF is derived from corn cobs and oat hulls [14]. According to Choi et al. [15], the global production of corn-grain has increased by 40% over the past decade and reached over 1 billion tons of production recently. This would enhance the production of corn residue which is stated about 47 to 50% of their residues are wasted [15]. On the other hand, it was reported that about 23 million tons of oat was globally produced in 2018 with oat hull waste representing 25 to 35% of the entire production [16]. Both of the residues need to be treated; hence, both of them have been recognized as safe and environmentally friendly solvents since they can be obtained from biomass feedstocks to which an exposure limit on humans up to 6.2 mg/day is permitted [17].

## 2. Results

### 2.1. Characterization of Crude Biodiesel

#### 2.1.1. Differential Scanning Calorimetry

**Figure 1:**
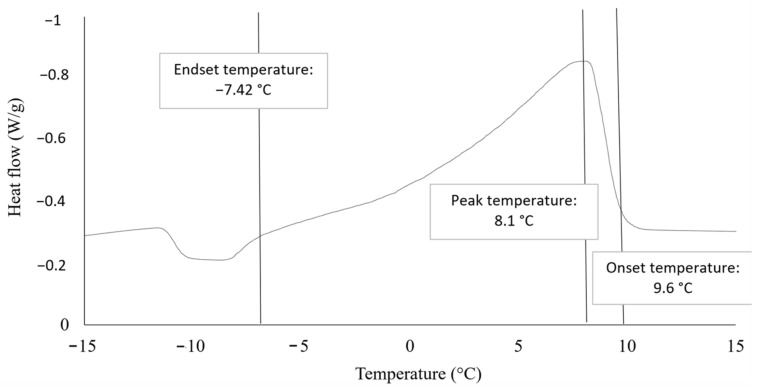
Graph of temperature vs. heat flow for biodiesel sample.

The DSC curve as in Figure 1 represents the temperature relationship on the heat flow as the outcome of calorimetric measurements for the biodiesel sample. The DSC graph demonstrated one exothermic peak, indicating the crystallization peak. The onset temperature is the temperature at which crystallization begins, the peak temperature indicates the temperature at which the maximum reaction rate occurs, and the end set temperature represents the temperature at which the process ends [12].

#### 2.1.2. Gas Chromatography–Mass Spectroscopy

**Figure 2:**
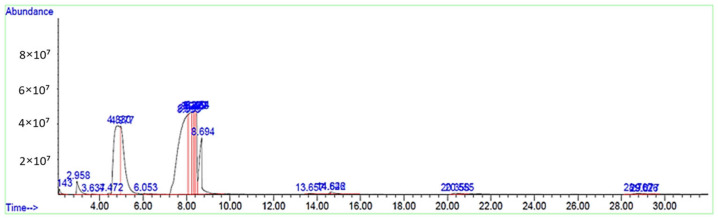
GC-MS chromatograph of biodiesel.

The crude biodiesel obtained after 24 h of gravity settling is analyzed using GCMS analysis to examine its quality in terms of FAME purity and the properties of biodiesel. Besides the sample of crude biodiesel, 16 biodiesel samples based on the different parameters for SAC had also been studied for GCMS characterization. The properties that can be obtained from the results are systematic name, retention time, correction area of individual components and the sum of the correction area. Figure 2 shows the abundance versus retention time graph for the chromatogram of GC-MS analysis for the crude biodiesel. 

### 2.2. Effect of Coolant Temperature in SAC

**Figure 3:**
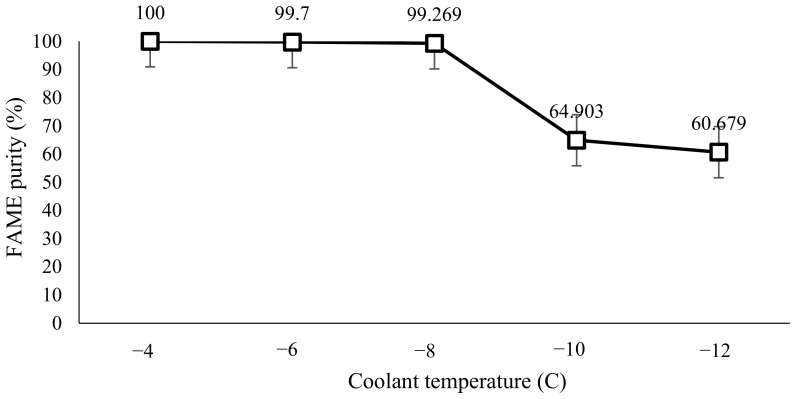
FAME purity against coolant temperature.

The cooling time and stirring rate were kept constant at 15 min and 140 rpm, respectively. The temperature of the coolant in the chiller is adjusted within the parameter range of the experiment. The parameter range for coolant temperature is −4 °C, −6 °C, −8 °C, −10 °C and −12 °C. The coolant used is a 50% (*v/v*) ethylene glycol solution with water [12]. Figure 3 shows the plotted graph using GC-MS data for FAME purity against coolant temperature while Table 1 showed the observation of the effect of coolant temperature in SAC. For the coolant temperature parameter, at a constant 140 rpm and cooling time of 15 min, a coolant temperature of −4 °C indicates the optimum value to yield the highest purity of FAME content which is 100% purity.

### 2.3. Effect of Cooling Time in SAC

**Figure 4:**
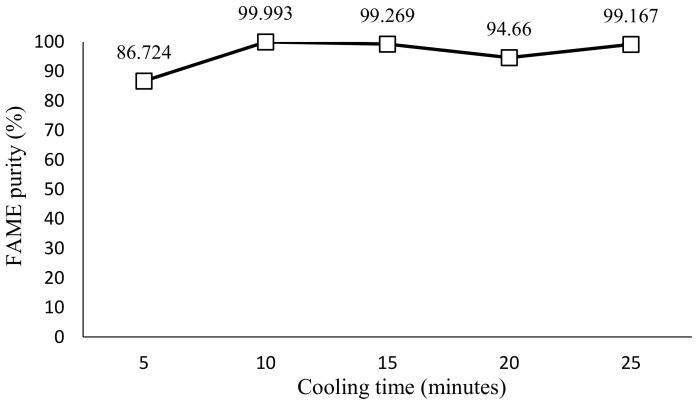
FAME purity against cooling times.

For this part of the experiment, the coolant temperature and stirring rate were kept constant, at −8 °C and 140 rpm, respectively. The parameter range for cooling time is 5 min, 10 min, 15 min, 20 min, and 25 min. Figure 4 shows the plotted graph using GC-MS data for FAME purity against cooling time, while Table 2 showed the observation of the effect of cooling time in SAC. For the cooling time parameter, at constant −8 °C and 140 rpm, a cooling time of 10 min indicates the optimum value to yield the highest purity of FAME content, which is 99.993% purity.

### 2.4. Effect of Stirring Speed in SAC

**Figure 5:**
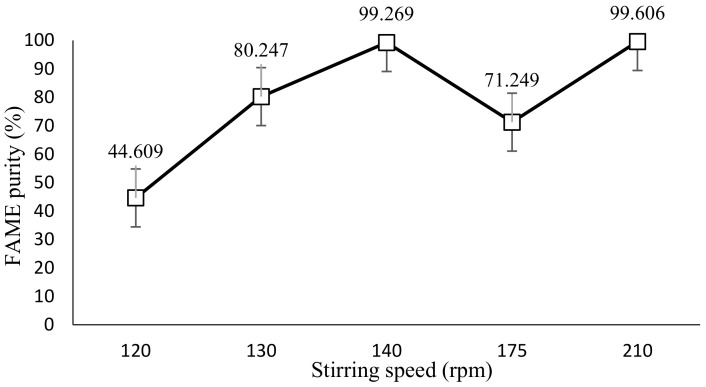
FAME purity against stirring speed.

For this part of the experiment, the coolant temperature and cooling time were kept constant, at −8 °C and 15 min, respectively. The parameter range for stirring speed is 120 rpm, 130 rpm, 140 rpm, 175 rpm and 210 rpm. Figure 5 shows the plotted graph using GC-MS data for FAME purity against stirring speed while Table 3 showed the observation of the effect of stirring speed in SAC. For the stirring speed parameter, at a constant −8 ℃ and cooling time of 15 min, a stirring speed of 210 rpm indicates the optimum value to yield the highest purity of FAME content, which is 99.606% purity.

## 3. Discussion

### 3.1. Characterization of Crude Biodiesel

#### 3.1.1. Differential Scanning Calorimetry

From Figure 1, the value obtained from the graph for onset temperature is 9.6 °C, the peak temperature is 8.1 °C and the end set temperature of biodiesel obtained is −7.42 °C. The temperature range for the following section of the experiment was determined using the SAC approach employing the crystallization point of biodiesel from the analysis. It is in line with the finding from a previous study conducted by Samsuri et al. [12], where the starting point of crude biodiesel crystallization was 9.45 °C, which was maximal at 8.4 °C, thus showing the highest rate of reaction. Towards the end, the temperature dropped to −5.18 °C, indicating the end of the experiment. 

#### 3.1.2. Gas Chromatography–Mass Spectroscopy

According to Figure 2, the total composition of FAME percentage obtained from the crude biodiesel is 99.12% with the total amount of unsaturated fatty acid and saturated fatty acid form at 58.54% and 40.76%, respectively. The fatty acid available in the crude biodiesel is Dodecanoic acid, Methyl tetradecanoate, 9-Hexadecanoic acid, Hexadecanoic acid, 9-Octadecenoic acid, Methyl strearate and Eicosanoic acid. The highest correction area obtained from an individual component is from 9-Octadecenoic acid. Table 4 shows the tabulated results for the systematic name (Library/ID), trivial name, types of fatty acids, retention time and the percentage of FAME composition for the crude biodiesel.

### 3.2. Effect of Coolant Temperature in SAC

The trend line in Figure 3 shows a slight decrease in trend, from −4 °C to −8 °C, until it drops downs steeply, at −10 °C, until −12 °C. The highest percentage of 100% purity is at the highest temperature, which is at −4 °C; meanwhile, at −10 °C, the FAME yield purity obtained is the lowest, which is at 60.68%. The crystallization temperature indicated by the onset temperature of this experiment is found to be at 9.6 °C and expected to end (as estimated) by the end set temperature of −7.42 °C. Hence, biodiesel is predicted to crystallize during conducting this experiment as all the parameters are lower than the crystallization temperature. As the coolant temperatures of −10 °C and −12 °C are much lower than the end set temperature, it is expected that this operating condition would yield a low purity of FAME. In comparison to biodiesel produced from solvent 1-butanol, the study mentioned that the highest biodiesel purity was achieved, 99.375%, when the coolant temperature was set at 12.7 °C [12]. This is because their onset and endset temperatures obtained from their DSC analysis were 9.45 °C and −5.18 °C, respectively, with a peak temperature of 8.4 °C. Nevertheless, this study was able to achieve even higher biodiesel purity which is 100% at a coolant temperature of −4 °C [12]. Hence, it is concluded that the use of 2-MeTHF as a solvent for SAC is able to produce higher biodiesel purity than 1-butanol despite the coolant temperature used. 

In reference to the FAME purity versus coolant temperature graph, a higher yield is obtained at a temperature farther than the end set temperature and closer to the crystallization temperature. This can be explained by Ahmad et al. [18], who explained that FAME is more likely to be trapped within the solid layer developed by glycerol and other contaminants when the such temperature is approaching the crystallization point. When the heat transfer rate is slower at higher coolant temperatures, the solid can form in a more orderly pattern, leaving the pure methyl ester to concentrate in the solution [19]. The solid development rate is larger at lower temperatures of coolant, resulting in more methyl ester retention into contaminating solids. This can be further proven by research from Yahya et al. [20], who stated that the rate of ice crystals or solid development is governed by the temperature of the coolant.

### 3.3. Effect of Cooling Time in SAC

The trend line from Figure 4 shows the FAME yield to be increasing from 5 min to 10 min, which is from 86.72% to 99.99% purity. From 10 min until 20 min, the FAME yield is found to decrease slightly before it increases at 25 min with the FAME yield of 99.17%. The highest purity obtained is at 10 min with 99.99% of FAME purity which can be considered to be pure biodiesel. Considering the result obtained from DSC, the crystallization temperature obtained is at 9.6 °C, and the end set temperature is at −7.42 °C. The experiment is carried out at a temperature close to the end set temperature, which is −8 ℃. As the experiment is conducted at a temperature much lower than the crystallization temperature, the solid layer from the contaminants is expected to be formed in the inner vessel. In comparison to biodiesel produced from solvent 1-butanol, the study mentioned that the highest biodiesel purity was achieved, 99.375%, when the cooling time was set to 35 min [12], which was longer than the optimum cooling time found in this study. The highest biodiesel purity found in this study is 99.99%, at a cooling time of 10 min, with which even higher biodiesel purity was obtained at a shorter cooling time compared to the study with 1-butanol. Hence, it is concluded that the use of 2-MeTHF as a solvent for SAC is able to produce higher biodiesel purity than 1-butanol despite the cooling time used.

In addition, it can be examined from Table 2 that during 5 and 10 min of cooling time, a white layer of glycerol is formed. Clear yellowish liquid biodiesel can also be seen formed in the vessel. Subsequently, during 15, 20 and 25 min of crystallization time, the liquid layer of biodiesel appears to be cloudy. The glycerol layer also appears to be thick over time. During 25 min of crystallization time, the layer of glycerol can be observed to be the thickest, resulting in a small volume of biodiesel formed. A larger yield of pure methyl ester was attained by using a prolonged cooling period [19]. In addition, as stated by Ahmad and Samsuri [11], for crystallization to occur, a longer crystallization time is preferable. However, as the FAME purity drops after an increasing amount of time, it can also be deduced that an increase in cooling time would also cause the growth of solid from the methyl ester to be reduced. The authors also stated that this may have been caused due to the saturation of the solute in the liquid phase inducing contamination of the solid. Consequently, the best range for cooling time is from 10 to 15 min, as proven by the purity of FAME at a constant temperature of −8 °C. Thus, this cooling time is not too prolonged for the separating process to take place.

### 3.4. Effect of Stirring Speed in SAC

Figure 5 shows an increase in the trend line from 120 rpm to 140 rpm from a value of 44.61% to 99.27%. At 175 rpm, the value of FAME purity decreases to 71.25%, and it increases to its highest purity at 210 rpm at 99.61%. In order to improve the formation of a solid, an aid for the solution movement is essential [18]. The parameter of this experiment is affected by the rate of stirring speed that is set by the laboratory mixer. As stated by Mohammed and Bandari [21], in maintaining a continuous temperature distribution and system flow, a gradual motion is essential. Therefore, a steady increase in stirring speed is chosen (120 rpm, 130 rpm and 140 rpm). After that, there is a disparity in stirring speed as the increment between the value is high (140 rpm, 175 rpm and 210 rpm). Consequently, this describes the irregularity of the trend line after 140 rpm. In comparison to biodiesel produced from solvent 1-butanol, the study mentioned that the highest biodiesel purity was achieved, 99.375%, when the stirring speed was set at 175 rpm [12]. Under similar stirring speeds, it is found that this study produced lower biodiesel purity (71.25%) compared to the one with 1-butanol. The highest biodiesel purity found in this study is 99.61% at a stirring speed of 210 rpm, which was a higher stirring speed used compared to the study with 1-butanol. Nevertheless, it is concluded that the use of 2-MeTHF as a solvent for SAC is able to produce higher biodiesel purity than 1-butanol despite the stirring speed used.

Furthermore, the efficiency of the purification can be observed from the graph by the purity of FAME. The highest value FAME yield can be seen at the highest stirring speed, which is 210 rpm. The contaminant in the biodiesel is circulated at a high flowrate, causing high separation between the solute and the solution. As Jusoh et al. [22] showed in their research, the formation of a high shear force, which could separate the solute from the solution, is imposed by a high circulation flowrate. Low separation is produced at a low stirring rate resulting in low purity of FAME because the solution moves more slowly. For the stirring rate of 175 rpm, there is a sudden drop in FAME purity. Although high stirring can yield good separation of contaminants, the moderate flow would also be prone to scrape away the solid developed on the vessel wall. This causes the impurities to mix with the liquefied biodiesel, resulting in low purity of FAME. This is researched by Mohammed and Bandari [21], who stated that stirring vigorously could prolong the solidification process and lower the liquid phase’s final concentration.

## 4. Materials and Methods

### 4.1. Materials Used

For this experiment, palm oil was purchased from a nearby supermarket. Meanwhile, methanol and KOH were obtained from the UTP laboratory. About 1000 mL of oil with 12.75 g of KOH as catalyst and 225 mL of methanol as solvent was used in the transesterification process. Meanwhile, for the SAC process, ethylene glycol and water were used as a coolant in the chiller. Crude biodiesel from the transesterification method that already completed the gravity settling process was used as feed for the SAC process. Assisting solvent, 2-MeTHF was added to the crude biodiesel for the purification process. 

### 4.2. Transesterification Process

**Figure 6:**
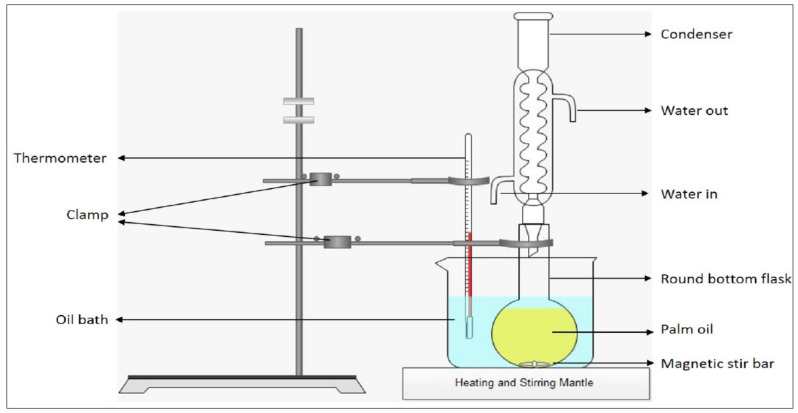
Transesterification method setup.

The experimental setup for the transesterification process is referred to the study conducted by Ahmad and Samsuri [11] as shown in Figure 6. To begin with, 1000 mL of palm oil was poured into a round-bottom flask. Next, the flask was heated at a reaction temperature of 60 °C, which is controlled by the heating mantle. At the same time, 12.75 g KOH was dissolved in 225 mL of methanol. After that, the solution of methanol and KOH was poured into the heated oil in the flask and stirred for 10 min. The product obtained from this transesterification process is known as crude biodiesel. Later, 1 mL of the product was extracted into a glass vial for DSC and GCMS analysis.

### 4.3. Solvent-Aided Crystallization

**Figure 7:**
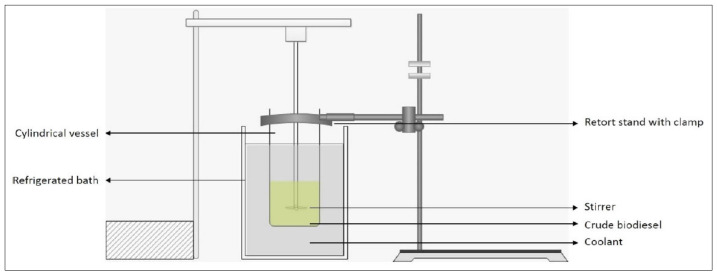
Solvent-aided crystallization method setup.

The experimental setup for the SAC process is referred to the study conducted by Ahmad and Samsuri [11] as shown in Figure 7. Firstly, the chiller was turned on to cool down the coolant temperature. The desired temperature was set before conducting the experiment. The range temperature for the whole experiment is between −4 °C and −12 °C. After that, about 500 mL of crude biodiesel with 1 wt.% of 2-MeTHF were fed into a cylindrical vessel (11 cm × 24 cm). The vessel was placed inside the chiller which was filled with coolant once the desired temperature was reached. Next, the stirrer was switched on and left until the expected cooling time. 

Solid contaminants are formed on the inner surface of a vessel, leaving pure biodiesel in liquid form. Subsequently, pure biodiesel was poured from the vessel to drain it out and detach the solid contaminants from the surface of the vessel. The solid contaminant was left to melt completely at room temperature. Thereupon, a sample of purified biodiesel was taken for GCMS analysis. The entire procedure was repeated under different operating conditions which are the temperature of the coolant, cooling time and stirring speed. The parameter range for coolant temperature is −4 °C, −6 °C, −8 °C, −10 °C and −12 °C, while cooling times are 5, 10, 15, 20 and 25 min, and stirring speeds are 123 rpm, 134 rpm, 140 rpm, 175 rpm and 210 rpm. All of the experiments were repeated twice, and average results were calculated for better data collection. 

### 4.4. Characterization of Biodiesel

#### 4.4.1. Differential Scanning Calorimetry

DSC is a device used to determine the amount of energy required to achieve a zero-temperature differential between a component and an inert reference substance by subjecting the two specimens to comparable temperature regimes in a contained manner [23]. The heat capacity or enthalpy of a sample of known mass is measured as changes in heat transfer. This analysis is suitable for glycerol and biodiesel, which are highly viscous melts [11]. 

Calibration of trials is used to record the temperature change and correlate it to the enthalpy change in the sample. The crude biodiesel sample is brought into equilibrium between −15 °C and 30 °C, at the rate of 5 °C min^−1^ for DSC measurement. In this research, it is vital to determine the crystallization point of the biodiesel sample to determine the lowest point of cooling temperature for the SAC process [11]. The crystallinity of materials is linked to the change in enthalpy by the energy required from the melting transition to proceed [23].

In this study, in DSC analysis, the sample was equilibrated, at 30 °C, and cooled immediately, at −15 °C, at a rate of 5 °C min^−1^. Afterwards, the sample was maintained for 1 min and heated to 30 °C at a rate of 5 °C min^−1^. Therefore, the procedure for transesterification was now complete. The remaining crude biodiesel was further used for gravity settling for 24 h before proceeding with the DSC analysis. 

#### 4.4.2. Gas Chromatography–Mass Spectroscopy

GC-MS is an analytic technology which combined gas–liquid chromatography separation features with mass spectrometry detection techniques to identify distinct compounds inside a test sample [24]. The mass of the analyte fragments is being used to identify these compounds. In academic research, this device facilitates the characterization and detection of newly synthesized or derivatized compounds by studying the new components [24]. Retention time (RT) is the time required for the compound to pass through the injection port to reach the detector [25]. 

In this study, the GC-MS device used in this experiment is PerkinElmer Clarus 600 Gas Chromatograph (GC). A flame ionization detector (FID) and an Elite 5-MS column with a dimension of 30 m × 250 µm × 0.25 µm of film thickness were installed in the GC. This device is used twice in this experiment, once after the reaction of transesterification (initial content of biodiesel) and lastly after conducting SAC (final content of biodiesel). During GCMS analysis, the oven temperature was set at 150 °C and held for 1 min. Afterwards, the temperature was raised to 240 °C, at 5 °C min^–1^ ramping speed, and was maintained for 5 min.

To determine the biodiesel purity, the percentage composition of individual FAME was computed using the following Equation (1). The biodiesel purity computation was then performed for all prominent peaks. For each SAC trial run as well, GC-MS would be used to determine the yield of FAME over all purified biodiesel samples. The purified biodiesel was left to melt after being treated to SAC and was collected for GC-MS analysis to determine the percentage of FAME composition to define its purity using the same mentioned formula.

(1) $$\mathrm{Percentage}\;\mathrm{composition}\;\mathrm{of}\;\mathrm{FAME}\;(\%)\;=\;\frac{\mathrm{Peak}\;\mathrm{area}\;\mathrm{of}\;\mathrm{individual}\;\mathrm{component}}{\mathrm{Summation}\;\mathrm{of}\;\mathrm{correction}\;\mathrm{area}}$$

## 5. Conclusions

Biodiesel is a non-toxic and biodegradable diesel alternative that is synthesized by the process of transesterification. 2-Methyltetrahydrofuran (2-MeTHF) can be used in chemical synthesis as an alternative to organic solvents for this project. In this context, it is used to replace solvent 1-butanol from a conducted previous study. The process parameters for SAC, which are different coolant temperatures, cooling time and stirring speed, are studied and analyzed for optimization. The chemical composition of biodiesel was taken into account when purified by the SAC process. The optimization process is considered successful once the optimum parameter value produces the highest purity of biodiesel, thus indicating that the biodiesel is free of contaminants. The optimum value to yield the highest purity of FAME for parameters coolant temperature, cooling time and stirring speed is −4 °C, 10 min and 210 rpm, respectively. Hence, by the proposed optimize parameter, it can be taken into account that SAC is effective in the purification of biodiesel. In conclusion, experimental research on the SAC method can assist in improving biodiesel purification. 

For future study, a techno-economic feasibility study (TEFS) and cost benefit analysis will be carried out in order to estimate the cost as well as energy for this SAC system for implementation in industrial applications. The energy cost of the process can vary depending on factors such as the source of the feedstock, the type of equipment used (refrigerated, stirrer), and the efficiency of the process. Additionally, the benefits of the biodiesel purification strategy, such as reducing greenhouse gas emissions and decreasing dependence on fossil fuels, may outweigh the energy costs. The analysis would be needed to determine whether the biodiesel purification strategy is a worthwhile endeavor. In addition, process simulation software will be used for the determination of the scale-up process, including the equipment’s size, design, operation, and process parameters optimization. 

## Figures and Tables

**Table 1:** Observation for the effect of coolant temperature in SAC.

Parameter			Diagram	Observation
Temperature (°C)	Stirring Speed (rpm)	Cooling Time (min)		
−4	140	15	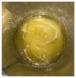	The glycerol layer is not completely crystallized.
−6			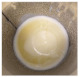	The colour of the biodiesel layer appears to be cloudy.
−8			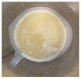	
−10			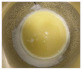	The biodiesel layer appears to be viscous. Only a little biodiesel is obtained. The glycerol layer appears to be thick.
−12			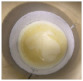	

**Table 2:** Observation for the effect of cooling times in SAC.

Parameter			Diagram	Observation
Cooling Time (min)	Stirring Speed (rpm)	Temperature (°C)		
5	140	−8	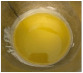	Glycerol is crystallized. A thin white layer of glycerol is formed.
10			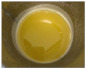	Glycerol is crystallized. A white layer of glycerol is formed.
15			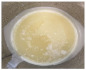	The colour of the biodiesel layer appears to be cloudy.
20			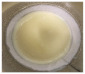	The colour of the biodiesel layer appears to be cloudy. The glycerol layer appears to be very thick.
25			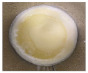	

**Table 3:** Observation for the effect of stirring speed in SAC.

Parameter			Diagram	Observation
Stirring Speed (rpm)	Cooling Time (min)	Temperature (°C)		
120	15	−8	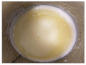	The colour of the biodiesel layer appears to be cloudy. The glycerol layer appears to be very thick.
130			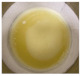	The pale colour of the biodiesel layer is formed. A thin white layer of glycerol is formed.
140			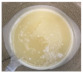	The colour of the biodiesel layer appears to be cloudy.
170			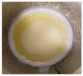	The biodiesel layer appears to be viscous. The glycerol layer appears to be thick. The colour of the biodiesel layer appears to be cloudy.
210			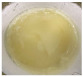	The biodiesel layer appears to be very viscous. Only a little biodiesel is obtained. The glycerol layer appears to be thick. The colour of the biodiesel layer appears to be cloudy

**Table 4:** Data of GC-MS results for crude biodiesel.

Systematic Name	Trivial Name	Types of Fatty Acid	Retention Time (min)	Composition of FAME (%)
Dodecanoic acid	Lauric	Saturated	2.143	0.41
Methyl tetradecanoate	Myristic	Saturated	2.959	1.6
9-Hexadecanoic acid	Palmitoleic	Unsaturated	4.473	0.14
Hexadecanoic acid	Palmitic	Saturated	4.828	18.31
Hexadecanoic acid	Palmitic	Saturated	4.977	12.19
Hexadecanoic acid	Palmitic	Saturated	6.053	0.27
9-Octadecenoic acid	Oleic	Unsaturated	8.321	48.64
9-Octadecenoic acid	Oleic	Unsaturated	8.376	3.19
9-Octadecenoic acid	Oleic	Unsaturated	8.456	6.48
Methyl strearate	Stearic	Saturated	8.692	6.86
Eicosanoic acid	Arachidic	Saturated	14.641	0.61
Hexadecanoic acid	Palmitic	Saturated	20.358	0.42
Total Unsaturated Fatty Acid				58.45
Total Saturated Fatty Acid				40.67
Total Fatty Acid				99.12

## Data Availability

Not applicable.

## Sample Availability

Samples of the compounds, if available, are available from the authors.
